# Ectopically expressed *Slc34a2a* sense-antisense transcripts cause a cerebellar phenotype in zebrafish embryos depending on RNA complementarity and Dicer

**DOI:** 10.1371/journal.pone.0178219

**Published:** 2017-05-18

**Authors:** Monica J. Piatek, Victoria Henderson, Amy Fearn, Bill Chaudhry, Andreas Werner

**Affiliations:** 1 RNA Interest Group, Institute for Cell and Molecular Biosciences, Newcastle University, Newcastle upon Tyne, United Kingdom; 2 Institute of Genetic Medicine, International Centre for Life, Newcastle University, Newcastle upon Tyne, United Kingdom; University Zürich, SWITZERLAND

## Abstract

Natural antisense transcripts (NATs) are complementary to protein coding genes and potentially regulate their expression. Despite widespread occurrence of NATs in the genomes of higher eukaryotes, their biological role and mechanism of action is poorly understood. Zebrafish embryos offer a unique model system to study sense-antisense transcript interplay at whole organism level. Here, we investigate putative antisense transcript-mediated mechanisms by ectopically co-expressing the complementary transcripts during early zebrafish development. In zebrafish the gene *Slc34a2a* (Na-phosphate transporter) is bi-directionally transcribed, the NAT predominantly during early development up to 48 hours after fertilization. Declining levels of the NAT, *Slc34a2a*(as), coincide with an increase of the sense transcript. At that time, sense and antisense transcripts co-localize in the endoderm at near equal amounts. Ectopic expression of the sense transcript during embryogenesis leads to specific failure to develop a cerebellum. The defect is RNA-mediated and dependent on sense-antisense complementarity. Overexpression of a *Slc34a2a* paralogue (Slc34a2b) or the NAT itself had no phenotypic consequences. Knockdown of Dicer rescued the brain defect suggesting that RNA interference is required to mediate the phenotype. Our results corroborate previous reports of *Slc34a2a*-related endo-siRNAs in two days old zebrafish embryos and emphasize the importance of coordinated expression of sense-antisense transcripts. Our findings suggest that RNAi is involved in gene regulation by certain natural antisense RNAs.

## Background

Long non-coding RNAs (lncRNAs) play an essential role in coordinating the spatio-temporal transcription of complex genomes. Natural antisense transcripts (NATs) constitute a particular group of lncRNAs with the hallmark of sharing complementarity with related, protein-coding sense mRNAs [1–3]. As a consequence, co-expressed sense/antisense transcripts can hybridize and potentially feed into double stranded RNA (dsRNS) mediated pathways. The biological relevance of dsRNA intermediates is supported by the observation that sense and antisense transcripts are usually detected in the same RNA preparation [4] and also by recent reports focussing on specific bi-directionally transcribed genes. In vertebrates up to 72% of genomic loci show evidence of bi-directional transcription and potentially express NATs [5]. These are enriched in testis, particularly in haploid spermatids, but are also found in somatic cells of all tissues [6, 7]. Remarkably, gene arrangements that give rise to sense-antisense hybrids are significantly under represented on the mammalian X chromosome [8, 9].

As a consequence of base complementarity, NATs are potentially highly specific regulators of their related sense transcripts, through interference at the transcriptional level or the formation of dsRNA. Both inhibitory and stimulatory impacts of NATs on the expression of related sense transcripts have been described [5, 10]. Alike other lncRNAs, NATs can form complexes with chromatin modifying proteins to alter the accessibility of the specific locus, thus restricting or enhancing transcription [11, 12]. Aberrant NAT-expression was also shown to induce DNA methylation of the sense promoter and knockdown the cognate protein coding gene [13, 14]. Mechanisms involving dsRNA formation affect the half-life of the sense transcript by masking microRNA binding sites, AU-rich elements or trigger RNA interference [15–19]. In these cases, hybrid formation occurs at the 3’end of the sense transcript; conversely, 5’ complementarity may increase translation efficiency by masking out of frame initiation codons [20, 21]. Nevertheless, there is a striking discrepancy between the large number of antisense transcripts and the current understanding about the associated regulatory mechanisms.

The *Slc34a* gene family encodes epithelial phosphate (Pi) transporters and selected vertebrate paralogues are transcribed in both directions [22]. In human and mouse, for example, the NAT overlapping the *Slc34a1* gene arises from a downstream single exon gene (*PFN3/Pfn3*) and represents a spliced and poly-adenylated read-through transcript. *Slc34a* encoded proteins are predominantly expressed in intestine and in kidney and are regulated by parathyroid hormone, vitamin D3 and fibroblast growth factor 23, factors that are essential to balancing body Pi levels [23, 24]. In contrast to the well-established physiological function of *Slc34a* encoded proteins, the biological role of the NAT is largely hypothetical and may not be related to maintaining phosphate (Pi) homeostasis.

**Fig 1:**
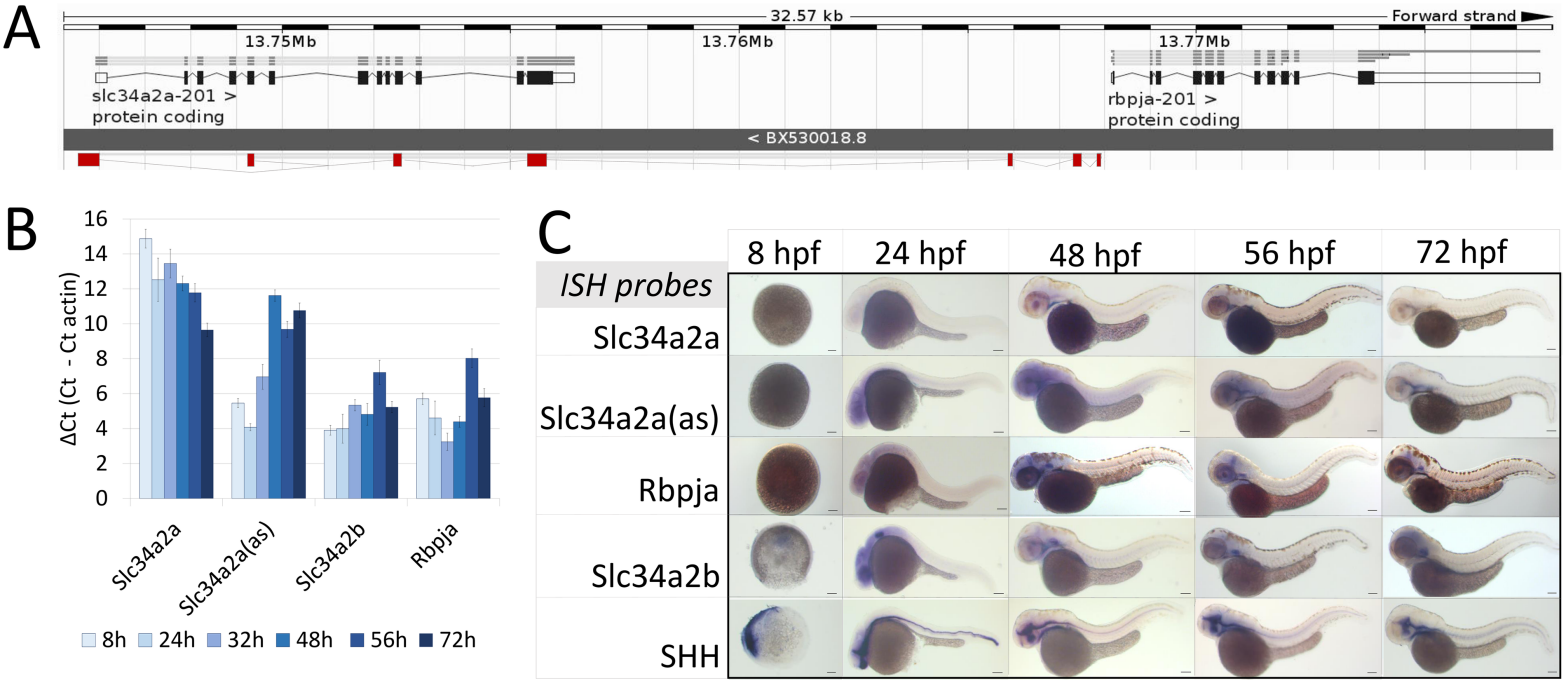
Expression of *Slc34a2a* and related transcripts during zebrafish embryogenesis. (A) Schematic representation of the *Slc34a2a*, *Slc34a2a*(as) and *Rbpja* loci. The antisense transcript *Slc34a2a*(as) is depicted in red. (B) RT-qPCR analysis of *Slc34a2a*, *Slc34a2a*(as) and *Rbpja* transcripts including the paralog *Slc34a2b*. Based on negative controls using RNA as an input, the detection limit was set at a ΔCt of 12 which is in agreement with ISH results. (C) Demonstration of *Slc34a2a*, *Slc34a2a*(as), *Rbpja*, *Slc34a2b* and *Shh* (Sonic Hedgehog) transcripts at progressing stages of development by whole mount ISH.

In zebrafish, the isoform *Slc34a2a* features a NAT which is driven by a bi-directional promoter shared with the *Rbpja* gene [22, 25] (Fig 1A). Due to the transparent appearance of zebrafish embryos, expression profiles of particular transcripts within the entire organism as well as the morphological consequences of gene overexpression or knockdown can be easily monitored. We have found previously that Slc34a2a sense and antisense transcripts are co-expressed in zebrafish embryos at around two days post fertilization. We also detected short RNAs by northern blotting during the period of co-expression [19, 26]. Here, we report that transcripts from the bi-directionally transcribed *Slc34a2a* locus show different spatio-temporal expression in whole mount zebrafish embryos. Only at hatching stage the protein coding sense transcript and the antisense transcript are co- expressed in the endoderm, coinciding with the detection of *Slc34a2a* related endo-siRNAs [19]. In order to explore the consequences of dysregulated sense-antisense co-expression and to characterize putative, antisense triggered regulatory mechanisms we injected various RNAs into fertilized zebrafish eggs and monitored the development of the embryos. Premature presence of the sense RNA leads to a specific phenotype that lacks the cerebellum. The defect depends on complementary RNA structures and can be rescued by the knockdown of Dicer.

## Results

To characterize potential interactions between complementary *Slc34a2a* sense and antisense transcripts, we quantified and visually demonstrated the expression of these RNAs during zebrafish development. We performed RT-qPCR with RNA extracted from individual zebrafish embryos and groups of five. The latter proved more reliable and therefore these data are presented. Primers specific for *Slc34a2a*, antisense, *Rbpja* and the paralogue *Slc34a2b* were used (S1 Table) and the Ct values were compared to the actin signal from the same cDNA sample. In line with previous end-point PCR data [26], we found a gradual increase of the *Slc34a2a* sense transcript with a parallel decrease of the antisense transcript (Fig 1B). The detection limit is around a ΔCt value of 12, suggesting that *Slc34a2a* is only expressed after about 2 days post fertilization (dpf). *Slc34a2b* is significantly expressed throughout embryonic development as was *Rbpja*, though the latter showed a different U-shaped pattern. The fact that the antisense transcript and *Rbpja* show significantly divergent expression suggest that the common promoter is directionally regulated. The two start sites are 229 bp apart and located at either end of a CpG island (Fig 1A). In order to assess the expression pattern, we performed whole mount *in situ* hybridization (ISH) of the genes assessed by RT-qPCR; *Shh* (Sonic hedgehog) was used instead of actin as a positive control. As detailed in Fig 1C the protein encoding sense transcript is not present during early developmental stages and only becomes detectable in the endoderm at 48 hpf. In contrast, the antisense transcript is expressed at early stages and is diffusely localized in the head and later becomes more confined to the pharynx, endoderm, as well as the primordial mid- and hindbrain channel. At early stages *Rbpja* mirrors the diffuse expression pattern of the antisense transcript, but from 48 hpf onwards, it localizes to the otic vesicle and outlines the posterior of the mesencephalon (midbrain). The transporter homologue *Slc34a2b* is expressed at early stages but only becomes defined after 48 hpf in the pharynx, endoderm and the otic vesicle. All embryos were destained extensively and diffuse signals were confirmed using published findings (*Rbpja*, [27]).

### Ectopic *Slc34a2a* sense RNA expression interferes with cerebellum development

**Fig 2:**
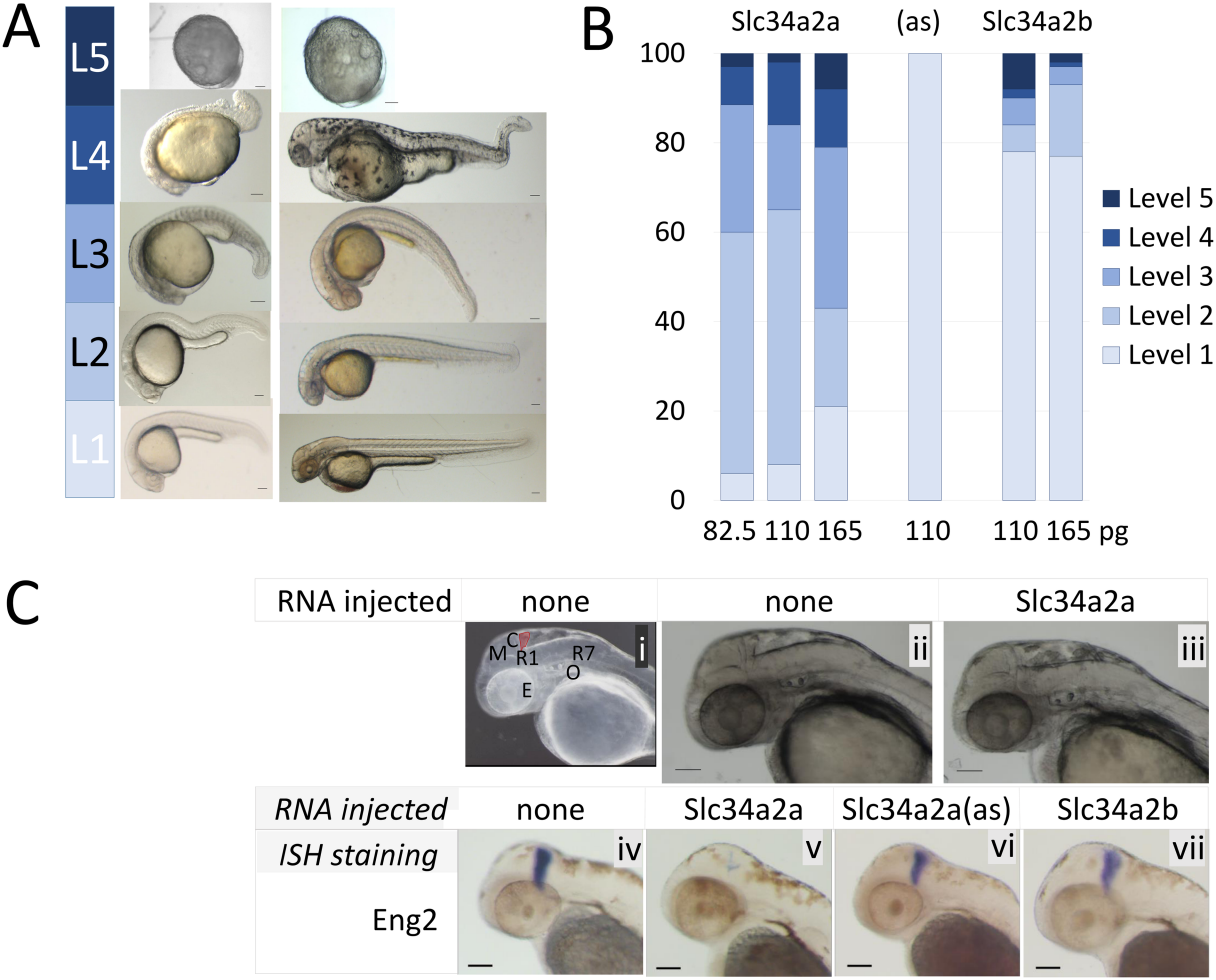
Injection of *Slc34a2a* RNA into fertilized zebrafish eggs. A) Visual classification of malformations depending on the severity of the defect: Level 1, wild type; level 2, one organ affected (size, shape or function, e.g. heart rate); level 3, 2–3 organs affected, level 4, multiple malformations; level 5, developmental arrest. B) Phenotypic characterization of zebrafish embryos injected with various RNAs, *Slc34a2a* (554 embryos in total), antisense (94 embryos) and *Slc34a2b* (538 embryos). C) (i) Anatomy of a 48 hpf zebrafish embryo: Y, yolk sac; E, eye; O, otic vesicle (ear); R1/R7, rhombomeres; M, mesencephalon; C, cerebellum, in red. (ii) non injected wild type embryo; (iii) *Slc34a2a* injected embryo; (iv) wild type embryo, Eng2 stained; (v) *Slc34a2a* injected embryo, Eng2 stained; (vi, vii) *Slc34a2a*(as) and *Slc34a2b* Eng2 stained.

Given the early expression of the antisense transcript and the later co-localization of the two complementary *Slc34a2a* transcripts, we tested the consequences of an ectopic presence of the sense transcript during early developmental phases by injecting *in vitro* synthesized RNA into fertilized eggs. To a certain extent this co-expression of sense and antisense transcripts would mimic the endogenous situation at 48hpf where we previously detected both transcripts by endpoint PCR and found endogenous siRNAs [26]. The paralogue *Slc34a2b* which shares 67.5% nucleotide sequence identity with *Slc34a2a* was used as a control. The embryos were classified for morphological abnormalities at 24 and 48 hpf into levels 1 (normal), 2 (one organ affected), 3 (2 or 3 visual/organ defects), 4 (multiple, severe defects) and 5 (developmental arrest) (Fig 2A). As demonstrated in Fig 2B, injection of exogenous *Slc34a2a* sense transcript interfered with the developmental program in a dose-dependent manner whereas injection of neither the antisense transcript nor *Slc34a2b* had significant adverse effects. The lower range of injected RNA (82.5 and 110 pg) produced a consistent, mild level 2 phenotype in more than 50 percent of the injected embryos, specifically lacking the cerebellum at 48 hpf. To demonstrate this defect more clearly embryos were stained for *Engrailed-2* (*Eng2*) expression, a specific marker for the cerebellum and mid-hindbrain boundary [28–30]. As shown in Fig 2C, the injection of *Slc34a2a* RNA eliminated the staining for *Eng2* almost completely, whereas control injections with antisense or *Slc34a2b* had no effect.

**Fig 3:**
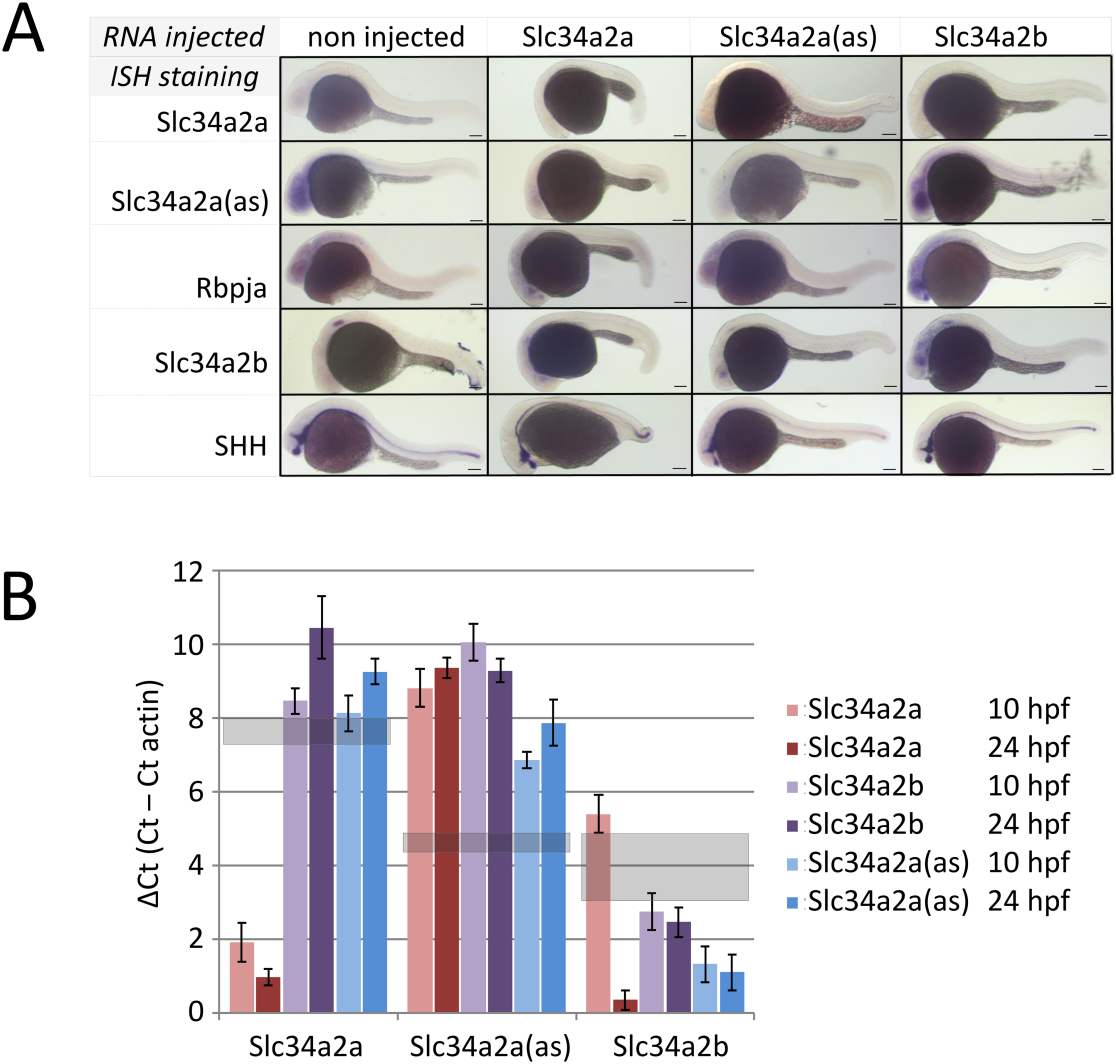
Injection of *Slc34a2a* RNA and related constructs into fertilized zebrafish eggs. A) ISH of wild type and injected embryos at 24 hpf. Horizontal labels at the top indicate the injected material, vertical labels, left, represent the probes used for ISH. B) RT-qPCR of injected zebrafish embryos; Slc34a2a, Slc34a2a(as) and Slc34a2b RNA was injected as indicated with the different colour from brown to blue and assayed after 10 and 24 hpf. The left group represents RT-qPCR reactions with Slc34a2a-specific primers; the middle group with Slc34a2a(as)-specific primers and the right group with Slc34a2b-specific primers. The values for non-injected controls are indicated with grey, transparent boxed.

To monitor the consequences of injections on the spatio-temporal distribution of *Slc34a* related transcripts we performed whole mount ISH with *Slc34a2a*, *Slc34a2a*(as) and *Slc34a2b* injected embryos at 24 hpf (Fig 3A). Remarkably, after injection of *Slc34a2a* the transcript remained undetectable, possibly the consequence of dsRNA formation with the endogenous NAT as this staining was also significantly fainter (Fig 3A, second column, first two rows). Injection of *Slc34a2a*(as) had an unexpected effect, a reduced staining of the antisense transcript (compare second row, first and third column), suggesting degradation and/or masking of both endogenous and exogenous *Slc34a2a*(as). These findings are in contrast to injections of the paralog *Slc34a2b* which produced an enhanced and more diffuse staining (column four, row four). The expression pattern of other genes (*Rbpja*, *Shh*) was unaffected by the injections (rows three and five). In contrast to the ISH results, RT-qPCR revealed a significant increase of sense transcript levels after injection of Slc34a2a whereas Slc34a2a(as) or Slc34a2b injection had no significant effect (Fig 3B, left panel). Remarkably, injection of all three transcripts (Slc34a2a, Slc34a2a(as) and Slc34a2b) caused a marked down-regulation of the antisense transcript, which is line with the ISH data (Fig 3B middle panel, Fig 3A, row 2). Injection of Slc34a2b RNA enhanced the RT-qPCR signal for Slc34a2b as expected whereas Slc34a2a(as) did not affect Slc34a2b levels. Slc34a2a injection appeared to stimulate Slc34a2b expression after 24 hpf (Fig 3B, right panel). The RT-PCR results are in line with the in-situ hybridizations but suggest that the experimental detection of transcripts may be affected by the interactions of injected and endogenous RNA (see Discussion). In the following experiments, the distinct cerebellar phenotype was used as a read-out to test details of the mechanism triggered by sense-antisense co-expression.

### RNA complementarity causes developmental phenotype

**Fig 4:**
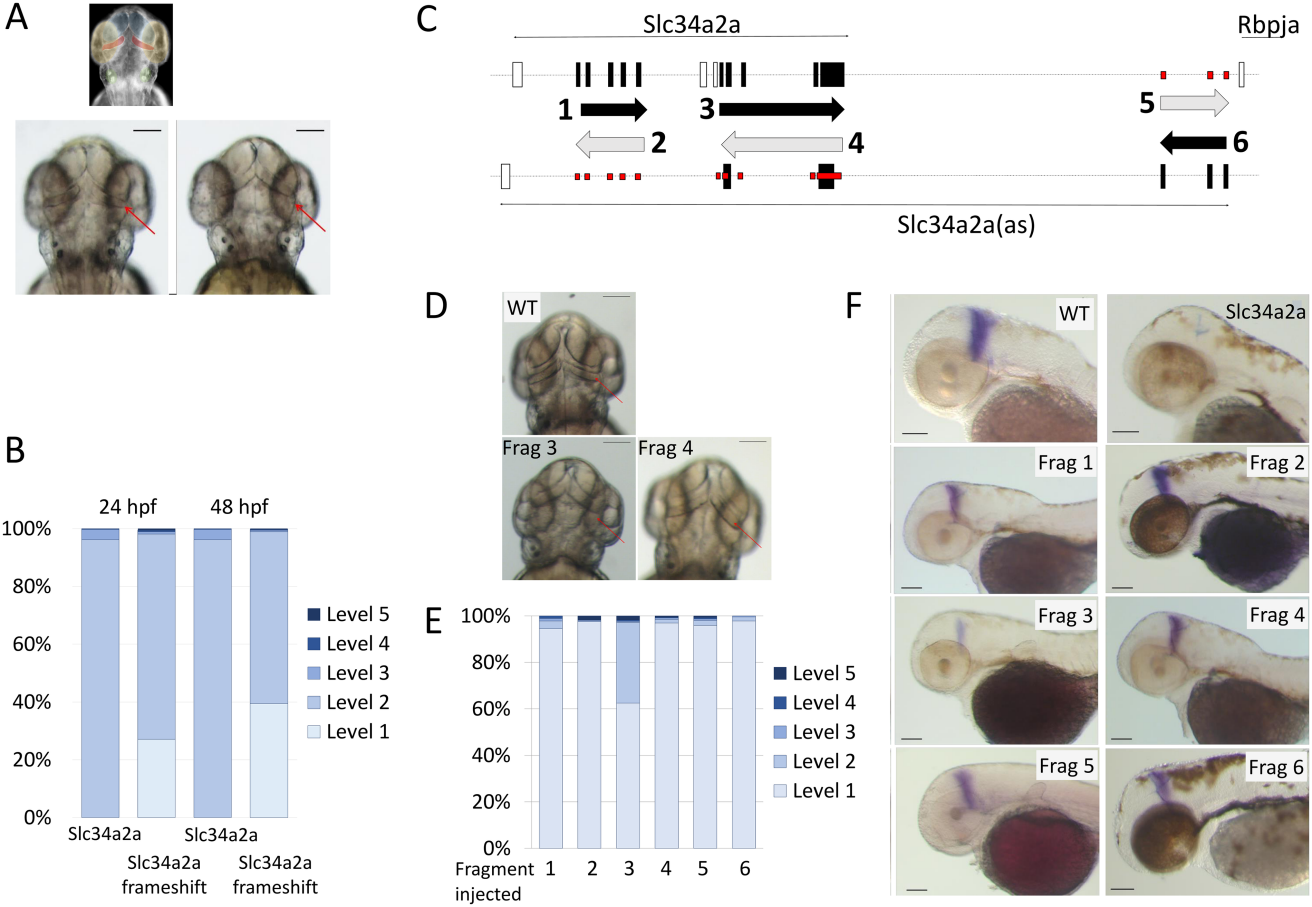
Injection of non- protein coding *Slc34a2a* RNA and *Slc34a2a* fragments interfere with zebrafish development. A) Schematic representation of a zebrafish head at 48 hpf; forebrain, blue; eyes, yellow; otic vesicles, green and cerebellum, red. Middle and left, wild type and *Slc34a2a*-FS injected embryo, respectively. Red arrows indicate the position of the cerebellum. B) Phenotypic quantification of *Slc34a2a* and *Slc34a2a*-FS injected embryos (364 *Slc34a2a*-FS injected embryos were assessed). C) Schematic representation of the fragments generated, even numbers represent sense orientation; uneven numbers, antisense orientation. The large black boxes represent exons comprised in the relevant fragments, the open boxes are exons that are not represented in the injected fragments. The small boxes in red indicate potential sites of hybridization of the injected fragments with an endogenous transcript on the opposite strand. D) Top view of 48 hpf embryos with the fragments (Frag) injected as indicated. E) Phenotypic assessment of injected embryos (90 or more per RNA). F) Eng2 stained embryos injected with the indicated fragments and the relevant controls. All the embryos were tested in parallel with the same solutions and under identical conditions to allow for a comparison of the relative intensities.

The injection of a protein coding transcript complementary to an endogenous antisense transcript can trigger various pathways that interfere with the developmental program of the embryos. Here, the specificity and the comparably subtle nature of the phenotype argues against an ‘RNA overdose’ effect [31]; however, both protein and RNA could be the active agent. A first hint towards an RNA-mediated mechanism was obtained when injection of capped and uncapped sense transcripts (which display different translation efficiencies) produced identical phenotypes. To rule out a protein mediated mode of action, we mutated the start codon to generate a *Slc34a2a* transcript with a frame shift (*Slc34a2a*-FS). Upon injection, a comparable phenotype as with the wild type sense transcript was observed, though with a slightly lower penetrance (Fig 4A and 4B). These results point to an RNA-mediated mechanism.

To narrow down the active region of the sense transcript, we generated fragments of both sense and antisense cDNAs with T7 and SP6 promoters at either end. We generated six transcripts (three in each sense and antisense direction, Fig 3C) and injected them into zebrafish embryos. The only fragment that caused phenotypic alterations of the cerebellum included the sequence of the sense transcript complementary to exons 4 and 5 of the antisense transcript (fragment 3 in Fig 3D–3F). None of the other fragments affected embryonic development, including the fragment complementary to exons 1–3 of the antisense transcript. *Eng2* staining confirmed the effect of fragment 3 on cerebellum development (Fig 3F). These findings suggest that it is not the interference with the antisense transcript per se but a sequence specific mechanism involving RNA complementarity that causes the developmental phenotype.

### Knockdown of *Slc34a2a*(as) using morpholino oligonucleotides

**Fig 5:**
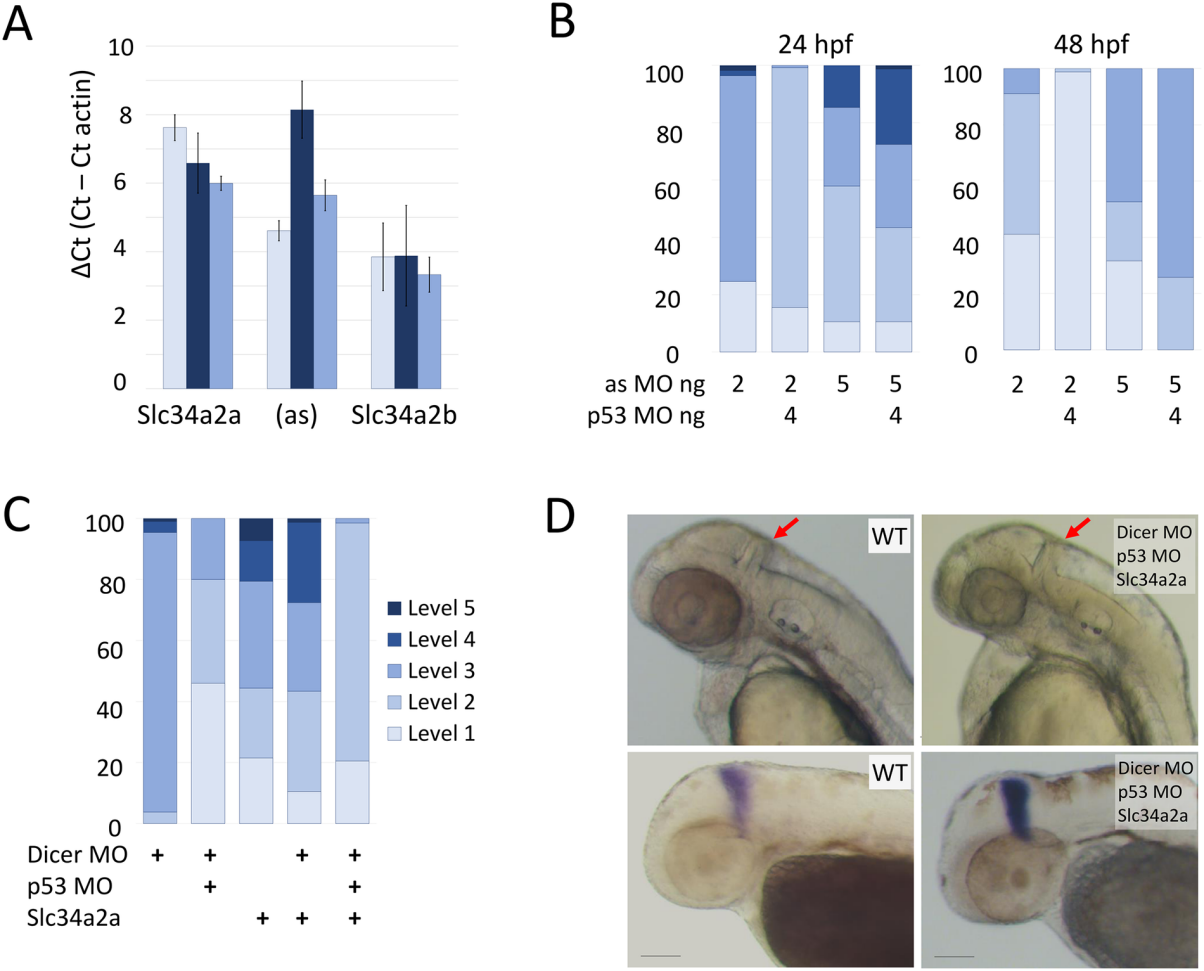
Morpholino knockdown of *Slc34a2a*(as) and Dicer. A) RT-qPCR quantification of *Slc34a2a*, *Slc34a2a*(as) and *Slc34a2b* after splice site morpholino injection at 24 hpf. Wild type non injected controls, light blue bars; 5 ng splice-site MO injected embryos are in dark blue; 5 ng scrambled MO injected embryos are in blue. B) Phenotypic characterization of MO injected embryos at 24 and 48 hpf. The injected oligonucleotides and quantities are indicated below the bars. Phenotypic scaling was performed as described in Fig 2. C) Rescue of cerebellum development by Dicer knockdown. Phenotypic assessment of embryos injected with combinations of Dicer MO, p53 MO and *Slc34a2a*. D) 48 hpf zebrafish embryos injected with Dicer MO, p53 MO and *Slc34a2a* as indicated in the pictures. In the upper panel, heads with red arrows indicating the cerebellum are shown; the lower panel shows ISH of embryos with an Eng2 probe.

To investigate the role of the antisense transcript further, we aimed to knockdown *Slc34a2a*(as) with a splice site morpholino oligonucleotide targeting the start of the third exon (S2 Table). RT-qPCR confirmed a marked decrease in *Slc34a2a*(as) in response to the morpholino injections at 24 hpf (Fig 5A). At 2 pg/embryo, injections produced a variety of level three phenotypes and the outcome could be improved to level 2 with a concomitant knockdown of p53 [32]. Unlike *Slc34a2a* injections, the morpholino knockdown did not produce a specific phenotype affecting the cerebellum. By 48 hpf transcript levels of *Slc34a2a*(as) returned to wild type levels and the embryos were phenotypically normal (level 1, Fig 5B right panel). At 5 pg/embryo the splice site morpholino proved toxic, producing varied and unspecific defects and the outcome could not be improved with concomitant knockdown of p53 (Fig 5B). Moreover, the embryos only marginally improved in fitness at 48 hpf. These findings suggest that the knockdown of the antisense transcript has no apparent developmental consequences. This assumption is supported by the fact that brain development is initiated before 24 hpf and the knockdown of the antisense transcript is most prominent during this period [33]. In addition, the phenotypes observed after morpholino injection are inconsistent and rather attributable to general toxicity than to the specific effect observed upon *Slc34a2a* RNA injection. The fact that high doses (110 pg) of exogenous antisense RNA are well tolerated also argues against a (dose-dependent) role of *Slc34a2a*(as) in cerebellum development. We cannot, however, completely rule out the possibility, that the antisense transcript plays a biological role between 24 and 48 hpf.

### RNA interference

To test the occurrence of potential RNA duplexes *in vivo*, we knocked down Dicer in sense transcript injected embryos. Two published anti-Dicer morpholinos were used including the previously mentioned p53 targeting morpholino to reduce toxicity (S1 Fig and S2 Table) [34]. Fertilized eggs were injected with a cocktail of *Slc34a2a* sense RNA, and morpholinos targeting Dicer and p53. As demonstrated in Fig 5, both Dicer MO and *Slc34a2a* caused the majority of embryos to be scaled at level two or higher and p53 co-injection considerably alleviated the severity of the malformations. Whereas the *Slc34a2a* injected embryos displayed the previously established cerebellar phenotype, the embryos receiving the triple cocktail showed light or no developmental alterations (Fig 5C). Importantly, all fish developed a cerebellum which was clearly visible and produced distinct Eng2 staining (Fig 5D).

We hypothesized that siRNAs produced by Dicer could be loaded onto Argonaute proteins and mimic the action of microRNAs. A Blast search including the complementary regions of *Slc34a2a* sense and antisense transcripts identified three genes that showed significant seed identity including Rasl11b, Wnt4b and Psen2. We designed hairpin oligonucleotides containing a T7 RNA polymerase promoter sequence including the relevant sense-antisense fragments separated by a 5 base linker sequence (S3 Table). The hairpin RNAs were synthesized *in vitro* and injected alone (82.5 and 165 pg/embryo) or in combinations (40 pg each) into fertilized eggs. The phenotypes of the developing embryos were classified as described (Fig 2A). Only comparably high levels of RNA (165 pg/embryo) produced minor phenotypes with Rasl11b and Psen2. Ras11b injected level 2 embryos appeared normal and in perfect proportion but were slightly smaller than non-injected controls (hence level 2 classification; S2 Fig), however, this defect resolved by 48 hpf. Psen2 hairpins caused a specific brain phenotype with increasing amounts of RNA injected. In contrast to *Slc34a2a* sense injected embryos which showed a cerebellum specific phenotype, the hindbrain was more broadly affected after Psen2 hairpin injections with enlarged rhombomeres but minimally affected cerebellum. Combinations of low amounts of the different hairpin RNAs (40 pg each/embryo) failed to produce a developmental phenotype, hence there was no obvious synergism between the three different hairpin constructs. Above described experiments have an additional read-out: The sequence similarity of the three hairpin RNAs with Slc34a2a implies that the antisense transcript which is expressed during early embryogenesis represents a perfect target. The lack of a phenotype with all hairpin RNAs including an siRNA pool, again, argues against an essential role of *Slc34a2a*(as) in cerebellum development.

## Discussion

We have used zebrafish embryos to comprehensively map *Slc34a2a* sense and antisense transcript expression and to investigate the consequences of ectopic expression of the sense transcript encoding an epithelial Na/phosphate cotransporter. Natural antisense transcripts not only harbour large regulatory potential but also pose formidable technical challenges. For example, simulated co-expression of a sense-antisense transcript pair almost inevitably leads to altered expression levels of the endogenous transcript with little predictive value for an antisense RNA mediated regulatory mechanism. Moreover, general procedures such as RNA extraction in the presence of guanidinium salts (as contained in Trizol, for example) have profound effects on the experimental outcome by very efficiently promoting RNA hybridization [35]. As a consequence, sense and antisense transcripts expressed in different cells or cellular compartments will hybridize during RNA extraction despite being kept apart under natural circumstances.

The biological role of most natural antisense transcripts is still largely speculative and a variety of molecular mechanisms have been put forward (see reviews [36, 37]. Several lines of independent evidence suggest that dsRNA formation is an essential step in an antisense mediated regulatory cascade [18, 22, 38]. Hence, the expression of complementary transcripts must be tightly coordinated to enable a timely interaction and also to avoid ectopic formation of dsRNA, an event that potentially triggers an antiviral response [39]. We found widespread expression of the *Slc34a2a* antisense transcript in the head region at stages where the protein coding sense transcript was not expressed and predominant co-expression in the endoderm thereafter; although the antisense transcript remained expressed at significant levels in other tissues. This pattern confirms earlier results by Nalbant et al. who tested the expression of *Slc34a2a* transcripts in adult fish and found the transporter expressed in intestine, kidney and the eyes, whereas the antisense transcript was detected in all other tissues [40]. The co-expression of sense and antisense transcripts at 48 hpf implies that dsRNA can be formed. A report by Carlile et al. demonstrating *Slc34a2a* derived endo-siRNAs indirectly supports the presence of sense/antisense duplexes [19]. We attempted to demonstrate these molecules using the dsRNA specific antibody J2 followed by RT-PCR. We validated the protocol for cell lysis and immune purification of RNA duplexes using hybridized *Slc34a2a*/*Slc34a2a*(as) as spike-in probes that were analysed by RNAseq. In all eight samples tested the antibody retained > 90% of the dsRNA. The failure of detecting dsRNA *in vivo* is therefore likely due to the very low and transient nature of the RNA hybrid.

The *Slc34a2a* antisense transcript and *Rbpja* are driven from the same promoter but in opposite directions. The promoter contains a distinct CpG island which is frequently associated with the transcription of long upstream antisense transcripts (here in relation to *Rbpja*) [41]. Lepoivre et al. also found co-regulation of many CpG promoter driven sense/antisense transcript pairs, a feature that *Rbpja* and *Slc34a2a* antisense do not share. The two transcripts do show similar overall expression levels (Fig 1) but a distinctly different expression pattern, especially recognizable at stages past 24 hpf.

The ectopic expression of the protein coding sense transcript during early embryonic development causes a dose dependent phenotype that –at low doses- specifically affects the formation of the cerebellum (Fig 2). RNA injected into zebrafish eggs is distributed uniformly and, if coding, leads to an early expression of a transgene [42]. Considering the expression pattern of the antisense transcript, the complementary endogenous and injected *Slc34a2a*(as)/ *Slc34a2a* transcripts are therefore likely to hybridize in cells of the head region. dsRNA can be toxic and interfere with zebrafish embryonic development in a dose dependent manner, especially if more than 40 pg of dsRNA are injected per embryo [43]. The hybrids were found to trigger either the PKR-interferon pathway or saturate RNA interference and deprive the embryos from essential micro RNAs, mi430 in particular [44]. The latter scenario is particularly relevant if hairpin RNAs are injected, though up to 400 pg shRNAs were used in these experiments [45]. The observation that dsRNA derived from various genes produce significantly different phenotypes suggest a sequence specific mode of action, likely at low RNA concentrations [46]. Our findings that only the fragment encompassing the naturally occurring complementary region caused the brain phenotype, but not a different fragment of the same transcript, supports this conclusion. RNA concentrations used in our experiments are unlikely to cause unspecific effects since the amount of dsRNA form depended on the lowly expressed endogenous *Slc34a2a* antisense transcript. Moreover, the concentration of the shRNAs were chosen to mimic the maximal level of dsRNA formed between exogenous sense and endogenous antisense *Slc34a2a* transcript and were, with 165 pg/embryo below the amount used by Zhao et al. [47]. Hence, several lines of evidence corroborate the specificity of our experimental findings despite the reported drawbacks of RNA injections to manipulate zebrafish development. It has to be kept in mind though, that concentration gradients of injected material, RNA secondary structure and stability may significantly influences the local concentration of particular transcripts which in turn affect the development of the embryo.

Endo-siRNAs or microRNAs derived from long sense/antisense duplexes are found predominantly in plants and C. elegans but their occurrence has been disputed in vertebrate cells [48]. Recent findings, however, add weight to a hypothesis where sense/antisense derived dsRNA feeds into an RNAi mediated regulatory pathway. First, there are increasing numbers of reports demonstrating the co-expression of sense and antisense transcript in the same specific cell types [7]. This is in line with the ISH pattern detected in zebrafish embryos at 48 hpf (Fig 1) and also with the detection of endo-siRNAs from sense/antisense RNA at that stage [19]. Second, the involvement of Dicer in the processing of endogenous RNA-RNA hybrids was shown in both mouse brain and testis [18, 49]. Third, experiments using reporter constructs that are transcribed in both orientation point to a mechanism that involves dsRNA formation, dicer processing, siRNA formation and concomitant transcriptional silencing of the locus [50, 51]. The model systems included HeLa cells and zebrafish; of note, only in HeLa cells transgene related siRNAs could be detected [51], whereas in zebrafish dicer dependence of silencing and histone modifications were shown [50]. These findings suggest that the siRNAs generated through convergent transcription are difficult to detect, supported by the fact that even from transfected HeLa cells the siRNAs needed to be enriched with p19 to become detectable on northern blots [52]. We also attempted to detect Slc34a2a/Slc34a2a(as) related siRNAs by northern blot but could not detect a signal (not shown). With the assumption that endo-siRNAs can feed into RISC and eventually suppress the expression of target genes; we identified transcripts with complementarity to the Slc34a1 sense/antisense overlap (Rasl1b, Wnt4b and Psen2). Targeting Psen2 with a hairpin RNA affected the hindbrain but failed to produce the cerebellum specific phenotype. The possibility that a more comprehensive cocktail would create the phenotype related to ectopic expression of the entire sense transcript is conceivable, however, this was not the case here. Interestingly, minimal changes to RNA structure (frame shift, RNA truncation) that are not even close to the dsRNA region reduce the penetrance of the phenotype. Hence the structure and the bioavailability of the injected material play a pivotal role in shaping the outcome of the developmental program. The involvement of the three genes (Rasl11b, Wnt4b and Psen2) in producing the cerebellum phenotype is therefore debatable but may not be ruled out completely.

## Conclusion

We have demonstrated that ectopic expression of the Slc34a2a sense transcript during early zebrafish development leads to a specific brain phenotype and have used this system to get insights into RNA regulatory mechanisms. Dicer dependence of the phenotype and previous detection of endo-siRNAs suggest that both ectopic, as well as endogenous processing of sense –antisense pairs, involve an RNAi related mechanism. Our results emphasize the importance of correct timing of sense-antisense co-expression and support the involvement of RNA interference in antisense RNA mediated gene regulation related mechanisms. Intriguing possibilities for such action would include endo-siRNAs as microRNA-like posttranscriptional gene regulators or agents to establish a locus specific epigenetic status.

## Materials and methods

The cDNA clones used in this study have been published elsewhere, *Slc34a2a* and *Slc34a2a* (as) by Nalbant et al. [40], *Slc34a2b* by Graham et al. [53], Sonic Hedgehog (*Shh*) by Danesin et al. [54] and Engrailed 2 (Eng2) was a kind gift from C. Houart. Fine chemicals were purchased from Sigma Aldrich.

### Zebrafish (*Danio rerio*)

Zebrafish were housed under standard conditions on a constant 14hour on /10 hour off light cycle at 28C and fed with *Artemia nauplii* and commercial flake (Tetra) [55]. All animals were maintained according to the requirements of the Animals (Scientific Procedures) Act 1986 of the UK Government and conformed to Directive 2010/63/EU of the European Parliament under UK Home Office project licence 60/4548 held by BC. AB and Golden wild type strains (Zebrafish International Resource Centre) were used in these studies.

Embryos were collected in blue water (2 ml of 0.1% methylene blue to 1 L aquarium tank water) and after injection raised at 28°C in E3 (5.0 mM NaCl, 0.17 mM KCl, 0.33 mM CaCl, 0.33 mM MgSO, pH 7.0 with 1 M NaOH) or E3 PTU (0.2 mM 1-phenyl-2-thiourea (PTU) in E3). Solutions were changed every 24 hours. Staging of embryos was done in accordance to morphological criteria provided by Kimmel et al. [56].

### *In situ* hybridization (ISH)

Embryos to be used for ISH were collected at the appropriate stage and fixed overnight at 4°C in 4% PFA. If the embryos were older than 24 hours, then they were first dechorionated with 2 mg/ml pronase [55, 57]. If younger than 24 hours, embryos were fixed and dechorionated manually using forceps. Embryos were dehydrated using increasing concentrations of methanol in water (25%, 50%, 75%, and 100%) prior to storage at -20°C in 100% methanol.

Embryos were progressively rehydrated on ice with PBST and washed twice in 100% PBST. 10–20 embryos were treated in 10 mg/ml Proteinase K (Sigma, 1:1000 in PBST) at room temperature, treatment length was adjusted to the age of the embryos. Embryos were washed twice in PBST and post-fixed in 4% PFA for 20 min at room temperature. After two washes in PBST the embryos were hybridized at 65°C overnight in a hybridization mix containing (1% blocking reagent (w/v, Roche), 50% formamide, 25% 20x SSC, 0.1% yeast RNA (w/v, sigma), 0.01% (w/v, Heparin, Sigma), 0.1% Tween20, 0.1% CHAPS) plus the appropriate RNA probes.

The subsequent washes were completed at 68°C in 2x SSC for 5 min, 2x SSC for twice 30 min, 0.2x SSC for 2x 30 min and a final wash in 0.2x SSC at room temperature. Embryos were equilibrated in MAB (0.1% Tween20), blocked in MAB/2% blocking reagent (0.1% Tween20, 2 h) and incubated with anti-DIG antibody (Roche, 1:4000) in MAB/blocking reagent overnight at 4°C. The embryos were washed in MAB and PBST (0.1% Tween20) and then equilibrated in NTMT buffer (in mM: for 50 ml: 100 NaCl, 100 Tris HCl pH9.5, 50 MgCl_2_, 1% Tween 20). Developing solution consisted of 3.3 μl NBT (Roche) and 3.5 μl BCIP (Roche) per 1.5 ml NTMT buffer. Embryos were washed in PBST stored in 70% glycerol/PBS prior to imaging using a dissection light microscope (Leica). Groups within an experiment using the same probe were strictly handled in parallel to allow for a relative comparison of intensities.

### Injections

Borosilicate glass capillaries (Hilgenberg, Germany) were prepared using a pipette puller (Sutter Instrument Co). Morpholinos or RNA were rear-loaded and a Femtojet injector (Eppendorf) was used to consistently deliver a volume of 2 nl. Concentrations of RNA or morpholinos were altered to adjust dose. The morpholino oligonucleotides were designed and synthesized by GeneTools Inc. The sequences of the different morpholino oligonucleotides are given below.

### In vitro transcription

Plasmids were linearized using the appropriate restriction enzymes and capped mRNA was synthesized using the mMESSAGE mMACHINE kit (Thermo Fisher Scientific). Non-capped mRNA was made using either the T7 or SP6 MEGAscript Transcription kit (Thermo Fisher Scientific). All RNA was DNase treated and purified using SigmaSpin^™^ Reaction Clean-Up columns prior to use. RNA integrity was confirmed by gel electrophoresis. RNA was stored at -80°C until use. Prior to injection, RNA was diluted to the required concentration with 10% Phenol Red in water. The probes for *in situ* hybridization were generated as above but NTPs were replaced with a mix of DIG labelled nucleotides (Roche).

### RT-qPCR

Embryos to be used in RT-qPCR were not fixed but placed directly into Trizol (Ambion, Life Technologies) and stored at -80°C until needed. RNA from five embryos was extracted using 50 μl Trizol according to established protocols. The precipitated RNA was resuspended in 16 μl RNAse free water and treated with DNase I (Thermo Scientific) in the presence of MgCl_2_ and MnCl_2_. The reaction was stopped with EDTA and purified using SigmaSpin Reaction Clean-up Columns (Sigma Aldrich). Approximately 1 ug of total RNA was reverse transcribed using a Qiagen Omniscript RT kit and random nonamers (Sigma Aldrich); the supplier’s protocol was strictly followed. The cDNA was diluted 1:4 in RNAse free water whilst an aliquot of the corresponding RNA was diluted 1:10 as a negative control. 10 ul reactions were run with LightCycler^®^ 480 Sybr Green I Master mix and gene specific primers (PrimerDesign or Simga Aldrich) in 96-well plates. The Ct values of the reactions were compared to β-actin [58]. Each sample was run in triplicate and a minimum of three different clutches were analyzed for each data point.

## Supporting information

S1 Fig Knockdown of p53 alleviates morpholino toxicity at high concentrations. Non-target specific zebrafish embryo phenotypes can be caused by morpholino toxicity through the activation of p53 mediated apoptosis [59]. Therefore, a p53 morpholino was co-injected with the Dicer UTR morpholino to mitigate toxicity. Co-injection of 4 ng of p53 morpholino caused a partial rescue of 10 ng Dicer UTR injected samples. The rescue was only visible with high concentrations of Dicer UTR morpholino. When 4 ng p53 morpholino were co-injected with 2.5 ng Dicer UTR morpholino, 98.2% of embryos were classified as level 3. The predominant phenotypic feature were shorter body length and non-circular eyes.
(DOCX)

S2 Fig Injection of hairpin RNAs mimicking Slc34a2a/ Slc34a2a(as) interaction. A) Phenotypic classification of hairpin injected embryos at 48 hpf. B) Wild type, left; 165 pg Psen2, middle;165 pg Rasl11B, right. Red arrows indicate the space above the hindbrain, reduced in Psen2 embryos.
(DOCX)

S1 Table Primers used for qPCR experiments. (DOCX)

S2 Table Morpholino oligonucleotides, sequences and their respective targets. All morpholinos were designed by and ordered from Gene Tools LLC.
(DOCX)

S3 Table Hairpin RNA sequences. Sequence of the templates to synthesize hairpin RNAs encompassing the complementary sequence between Slce34a2a sense/antisense that is identical to the three candidate genes Psen2, Wnt4b and Rasl11b. T7 promoter sequence in red; Slc34a2a in pink; sequences in common between Slc34a2a sense, antisense and target gene in blue; spacer sequence in grey; *Xba*I restriction site in italic; M13 universal primer in purple.
(DOCX)
