# Supplementary figures and images for: Ectopically expressed Slc34a2a sense-antisense transcripts cause a cerebellar phenotype in zebrafish embryos depending on RNA complementarity and Dicer
Source: PLoS One. 2017 May 18;12(5):e0178219. doi: 10.1371/journal.pone.0178219 (PMC5436864; doi:10.1371/journal.pone.0178219)

S1 Fig


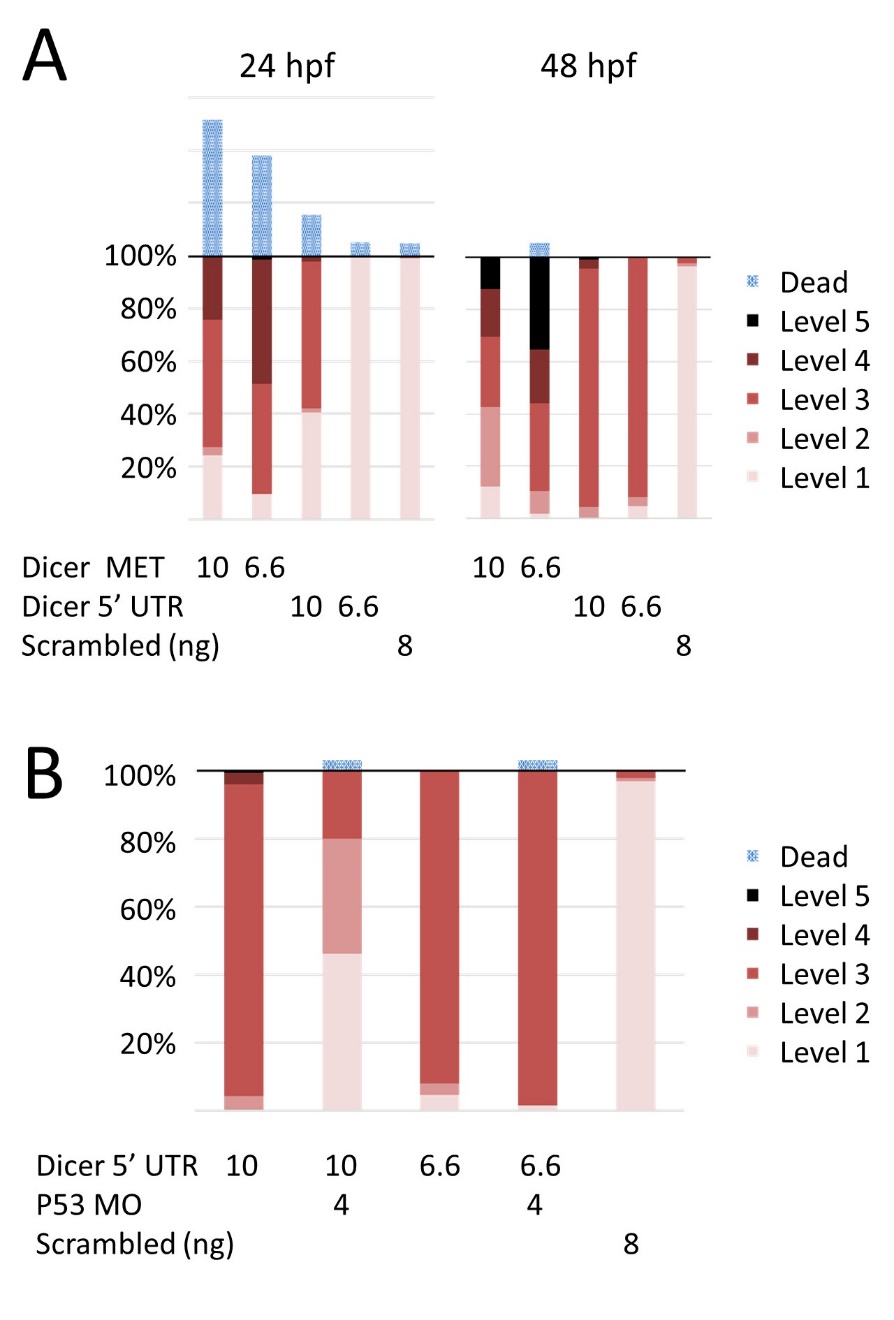

Supplement: S1 Fig — Non-target specific zebrafish embryo phenotypes can be caused by morpholino toxicity through the activation of p53 mediated apoptosis [59]. Therefore, a p53 morpholino was co-injected with the Dicer UTR morpholino to mitigate toxicity. Co-injection of 4 ng of p53 morpholino caused a partial rescue of 10 ng Dicer UTR injected samples. The rescue was only visible with high concentrations of Dicer UTR morpholino. When 4 ng p53 morpholino were co-injected with 2.5 ng Dicer UTR morpholino, 98.2% of embryos were classified as level 3. The predominant phenotypic feature were shorter body length and non-circular eyes. (DOCX) [file pone.0178219.s001.docx]

S2 Fig


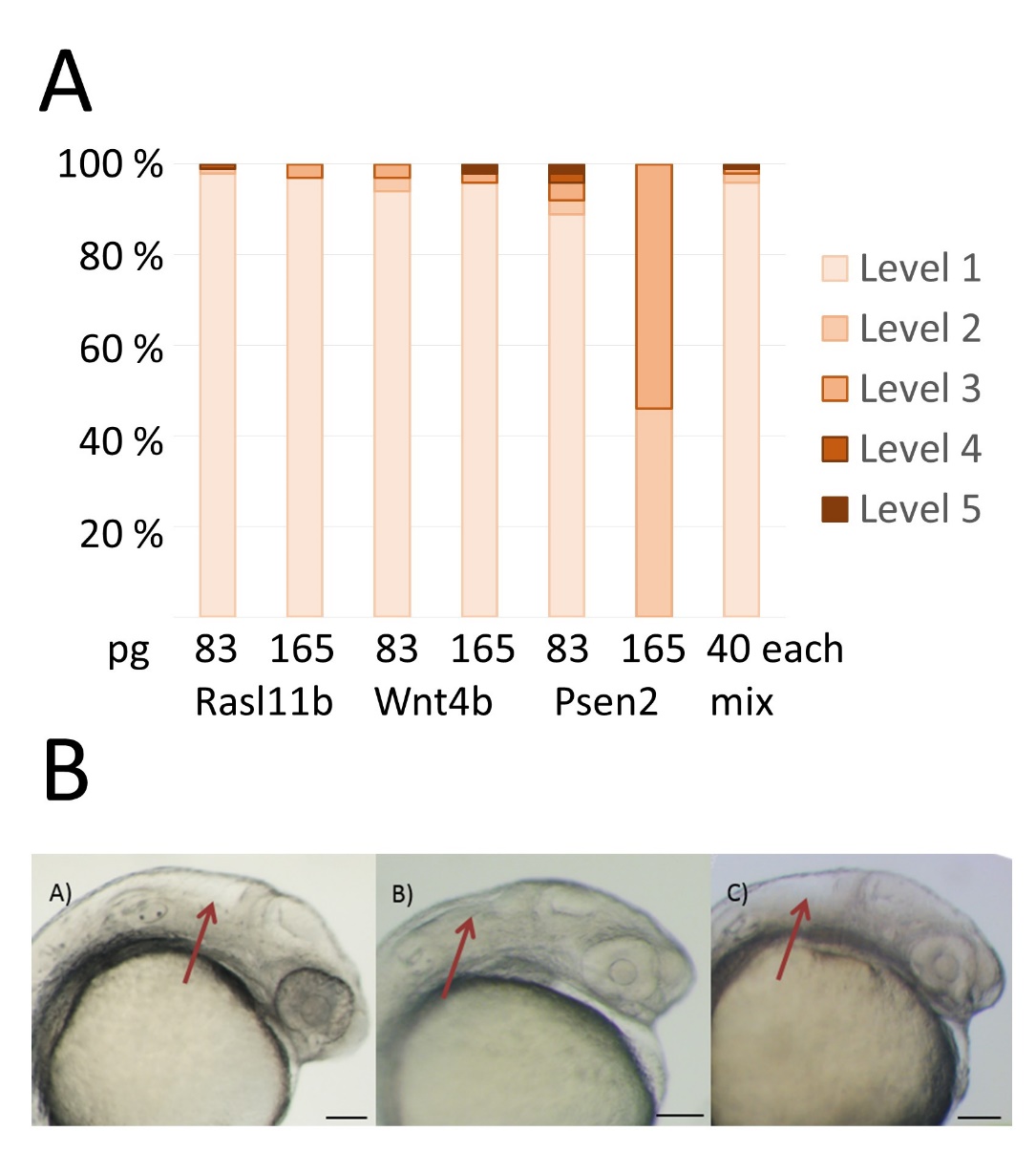

Supplement: S2 Fig — A) Phenotypic classification of hairpin injected embryos at 48 hpf. B) Wild type, left; 165 pg Psen2, middle;165 pg Rasl11B, right. Red arrows indicate the space above the hindbrain, reduced in Psen2 embryos. (DOCX) [file pone.0178219.s002.docx]
