# Supplementary material for: Ectopically expressed Slc34a2a sense-antisense transcripts cause a cerebellar phenotype in zebrafish embryos depending on RNA complementarity and Dicer
Source: PLoS One. 2017 May 18;12(5):e0178219. doi: 10.1371/journal.pone.0178219 (PMC5436864; doi:10.1371/journal.pone.0178219)
Supplement: S1 Table — (DOCX) [file pone.0178219.s003.docx]

S1 Table

| **Target transcript** | **Sequence** | **Product Length** |
| --- | --- | --- |
| Slc34a2a | Forward:  5’-AACACAGATTTCCCT TTTCCATTT- 3’  Reverse:  5’-CAAGAGGAGTTATAGC CGATGT-3’ | 121 |
| Slc34a2a | Forward:  5’-CCTGGGAAATGATGG AGCTGCAGG-3’  Reverse:  5’-CTCCAACAAGCTGGA AAGCTGAGC-3’ | 174 |
| Slc34a2b | Forward:  5’-ATCTGGCGGTGGGT TTGA- 3’  Reverse:  5’-CAGCATGGAGTTCAG GAGTTT -3’ | 92 |
| Slc34a2b | Forward:  5’-CCACTGGAGACCCTGC TGCC-3’  Reverse:  5’-AGCACAGGGCGTCCG GAGTG-3’ | 126 |
| Antisense | Forward:  5’-GAGAGTGGGGACG GACGGTTG- 3’  Reverse:  5’- CAGGAGTCCCAG CACACTGCAGA -3’ | 144 |
| Rbpja | Forward:  5’-CTGTCTCTCTTTGTT GTGGTTCT- 3’  Reverse:  5’-GCATTGATCCTGTGG TATCTTGA -3’ | 96 |
| Actin | Forward:  5’-CCGTCTTCCCCTCCA TCGTTGG -3’  Reverse:  5’-GTTGGTAATGATGCC GTCC TCGATG -3’ | 149 |
