# Supplementary material for: Ectopically expressed Slc34a2a sense-antisense transcripts cause a cerebellar phenotype in zebrafish embryos depending on RNA complementarity and Dicer
Source: PLoS One. 2017 May 18;12(5):e0178219. doi: 10.1371/journal.pone.0178219 (PMC5436864; doi:10.1371/journal.pone.0178219)
Supplement: S2 Table — All morpholinos were designed by and ordered from Gene Tools LLC. (DOCX) [file pone.0178219.s004.docx]

S2 Table

| Morpholino | Sequence | Target |
| --- | --- | --- |
| Antisense splice- site MO | 5’ – GCCATCTGGTGAAAAGACAGAGTTT – 3’ | Antisense pre-mRNA third exon |
| AS mismatch | 5’ – GACATCTCGTCAAAACACAGACTTT – 3’ | No target |
| Dicer 5’ UTR | 5’ – CTGTAGGCCAGCCATGCTTAGAGAC – 3’ | 5’ UTR of *dicer1* |
| Dicer start | 5’ – TCTTTCTCTTCATCTTCCTCCGATC – 3’ | Translational start site of *dicer1* |
| P53 | 5’ – GCGCCATTGCTTTGCAAGAATTG – 3’ | P53 |
| Standard control | 5' - CCTCTTACCTCAGTTACAATTTATA - 3' | No target |
