# Supplementary material for: Ectopically expressed Slc34a2a sense-antisense transcripts cause a cerebellar phenotype in zebrafish embryos depending on RNA complementarity and Dicer
Source: PLoS One. 2017 May 18;12(5):e0178219. doi: 10.1371/journal.pone.0178219 (PMC5436864; doi:10.1371/journal.pone.0178219)
Supplement: S3 Table — Sequence of the templates to synthesize hairpin RNAs encompassing the complementary sequence between Slce34a2a sense/antisense that is identical to the three candidate genes Psen2, Wnt4b and Rasl11b. T7 promoter sequence in red; Slc34a2a in pink; sequences in common between Slc34a2a sense, antisense and target gene in blue; spacer sequence in grey; XbaI restriction site in italic; M13 universal primer in purple. (DOCX) [file pone.0178219.s005.docx]

S3 Table

| **Gene** | **Overlapping sequence** | **Exon in Slc34a2a** | **Length (bp)** | **Orientation** | **Percent identity** |
| --- | --- | --- | --- | --- | --- |
| Psen2 | CTGCTGAATTC_ATGCTCAA | 10 | 20 | Forward | 95 |
| **TAATACGACTCACTATAGGG**TGAAGCTGCTGAATTCTATGCTCAAGGGACAAAAGTGTCCCTTGAGCATACAATTCAGCAGCTTCA*TCTAGAC*TGGCCGTCGTTTTAC | | | | | |
| Wnt4b | GAGCCTTTCGGACCT | 10 | 15 | Forward | 100 |
| **TAATACGACTCACTATAGGG**AACACGAGCCTTTCGGACCTGGCGGAAAAGTCCGCCAGGTCCGAAAGGCTCGTGTT*TCTAGA*CTGGCCGTCGTTTTAC | | | | | |
| Rasl11b | TGTTGCTGTTGCTGCA | 13 | 16 | Reverse | 100 |
| **TAATACGACTCACTATAGGG**CCCGCTGTTGCTGTTGCTGCAAGTGCAAAAGTGCACTTGCAGCAACAGCAACAGCGGG*TCTAGA*CTGGCCGTCGTTTTAC | | | | | |
